# Chloralamide

**Published:** 1893-06-24

**Authors:** G. Parker

**Affiliations:** Assist. Physician Bristol General Hospital


### New Drugs and Preparations.

[We should be glad to receive, at our office, 428, Strand, London, W.C., from the manufacturers, specimens of all new preparations and drugs which may be brought out from time to time.]

### CHLORALAMIDE.

By G. Parker, M.A., M.D., Cantab., Assist. Physician Bristol General Hospital.

One of the most fertile fields for study in recent years has been, and is still, the relation between the chemical composition of therapeutic agents and their physiological action. Organic chemistry, though able to tell us at present comparatively little of the reactions of substances while forming part of living bodies, has been able to manufacture a vast number of compounds whose effects on living bodies can be predicted to some extent beforehand from their composition. Thus a whole series of hypnotics and analgesics have been formed, and the qualities of each depend upon their position and rank in the series. If one member has certain valuable properties combined with some injurious ones, a higher or lower member of the series may be synthetically created, in which the valuable properties are, as it were, isolated. Hence we are slowly forming specialised instruments fit for definite therapeutic work, instead of looking for them haphazard among existing animal and vegetable products. The failures of these new agents, when tested in practice, are not so many as to seriously discredit the new method, and some drugs of undoubted value have been already discovered. Still the number which survive lengthy trials is not great. Among these survivors is the amide of chloral. If in the formic aldehyde $COH_2$ one of the H atoms is replaced by amidogen $NH_2$, formamide is the result, and when this is combined with another aldehyde, that of chloral, we get chloralamide. Of this we may say shortly that it possesses nearly the hypnotic value of chloral with hardly any of its dangers, and the claims which were made for it have been fairly substantiated by long and searching trials. We believe that the drug has passed from the experimental stage to the position of a servant on whose fidelity perfect reliance can be placed. It cannot, indeed, replace opium in combating pain, nor is it certain to throw into slumber any and every case on which it is tried, but while rather less powerful than sulphonal, or even chloral, its safety, pleasantness, and solubility render it much more manageable. At the Royal Infirmary, Bristol, to take one institution where it has been extensively used for some time, the usual dose is 20 to 30 grains, repeated once, if needful, after two hours. It is given in milk or suspended in mucilage, and no accident has ever occurred with it. In a case of tetanus in a boy aged seventeen under Dr. Shingleton Smith, ten grains were actually given every hour except during sleep for a week, and 100 grains daily for 25 days with quite satisfactory results, and even larger doses have been used. It is soluble in nine parts of water, especially if slightly acidulated, or in two parts of alcohol. Hence it can be administered in a glass of spirit and water at bedtime. It must not be given with alkalies or in hot liquids which easily decompose it. We have not been able to learn of any instances where a habit or craving has been produced by its use; and, on the other hand, it does not seem to lose its efficacy by continued administration. Compared with sulphonal, chloralamide is much more easily soluble, and more rapid in its action, less expensive, though a larger dose is necessary to produce the same effect, and there is less dulness and depression after the sleep is over.

Its great advantage over opium is its stimulant effect on the respiration, its negative effect on the secretions, and its safety in pneumonia, delirium tremens, and Bright's diseases. Here it may well replace the nauseous paraldehyde, as the best we can do for the delirium and wakefulness of acute pneumonia. In a case of aortic disease, in the later stages, where the patient could only snatch sleep propped upright and under the influence of morphia, chloralamide enabled her to lie down and rest without the opiate. W. V. Whitmare reports on fifty-two cases that sleep was obtained on average in an hour. There was no cardiac depression, and only in rare cases was there any gastric disturbance or cerebral after affects. He noticed especially its value in cardiac asthma and dyspnœa, and in phthisis where the sleep, cough, expectoration, and night sweats were greatly benefited. We must record, however, a few cases in which it has not merely failed to give sleep, but where unfavourable after effects followed, though these are very few indeed out of the enormous number in which it has been employed. No fatal case seems to be on record; Robinson mentions three instances of valvular disease, where a rapid and weak pulse followed its administration, and nine cases where abdominal pain occurred.

Osler and Toulmin mention various unpleasant results in sixteen cases, and G. E. Alford reports an instance where after thirty grains dissolved in spirit the patient suffered from stupefaction, faintness, semi-coma, and purging, and then fell into a prolonged sleep. W. B. Chapman, after giving a 45-grain dose to a robust patient, found the respirations run up to 124 in the minute, while the pulse was 105, and in another instance he observed eight hours of restlessness, headache, and vomiting, followed by a sleep of twelve hours. A few cases of transient eruptions and some of nausea are recorded, and R. Main had epistaxis and congestion of the face following each dose in a patient of eighty years with granular kidneys and other disorders. On the other hand, very many observers have never met with any drawbacks in a wide experience. In old age, chorœa, and in the sleeplessness of some forms of spinal disease and from overwork, it seems to be unrivalled. While it is useless in severe pain or violent mania, it may be administered with great advantage in rheumatic fever, neuralgia, tetanus, and epilepsy as a hypnotic. Lamphear advocates its employment after surgical operations in place of morphia, to give the patient a good night's rest after the shock.

A great mass of experiments have been made as to its action on animals by H. C. Wood, Cerna, Malaceowsky, and John Gordon. On comparing their results we find that chloralamide (*a*) acts chiefly on the cortex, and causes sleep by lessening its excitability; (*b*) it reduces to some extent the irritability of the cord; (*c*) ordinary doses stimulate respiration; (*d*) while excessive ones slowly reduce blood pressure.

Occasional idiosyncrasies may be met with in the employment of any drug, but we consider that few compare in safety with chloralamide. A little giddiness or nausea may sometimes occur, but the long experience at the Bristol Infirmary and elsewhere proves that for insomnia, accompanied by little or no pain, we have in chloralamide an active and practically harmless remedy.

